# The Second Law, Asymmetry of Time and Their Implications

**DOI:** 10.3390/e24070862

**Published:** 2022-06-23

**Authors:** Alexander Y. Klimenko

**Affiliations:** School of Mechanical and Mining Engineering, The University of Queensland, Brisbane, QLD 4072, Australia; a.klimenko@uq.edu.au

Explaining the asymmetry of the directions of time (the time arrow) is one of the major challenges for modern science. In the previous century, this discourse was led by philosophy, which strived to place antecedent causality at the very foundation of our thinking about the universe. While our understanding of time has been contributed to by philosophers and physicists alike (one may wish to mention Boltzmann, Russel, Reichenbach, Hawking, Davies, Zech, Penrose, Price and many other great minds), physics as a discipline shied away from the challenge of leading this discussion and preferred postulating causality in one form or another instead of trying to explain it. 

It seems, however, that this arrangement, which spanned over the whole century, is approaching its natural end: despite all its Sisyphean efforts, philosophy cannot define and explain antecedent causality in reasonably transparent terms and has to resort to invoking physical laws. This situation is hardly satisfactory from the cross-disciplinary perspective: antecedent causality is philosophically linked to the action of physical laws (most notably that of the second law of thermodynamics) while the same physical laws are conventionally justified and explained by postulating or implicitly presuming causality. It is more or less clear that this logical circle cannot be resolved by the power of the mind alone and needs physical experiments. While the reality of the time arrow is obvious at the macroscopic level, the ultimate challenge for physics is in identifying and detecting the fine mechanism that implements this arrow of time microscopically. From our perspective—that of physical realism—there must be a mechanism (e.g., “time primer”) that is responsible for the observed asymmetry of the direction of time and can be explicitly detected in experiments, at least in principle [1]. The cornerstone idea in creating this Special Issue is that physical sciences are bound to lead research into the direction of time, the second law of thermodynamics and their various implications. Macroscopic and microscopic considerations are connected by a fundamental result called the fluctuation theorem [2]. The fluctuation theorem demonstrates that, in the presence of antecedent causality, reversible microscopic dynamics such as Newtonian dynamics or quantum dynamics lead to asymmetric distributions of the values of fluxes in the system if it is out of equilibrium. 

The current special issue, “The Second Law and Asymmetry of Time”, involves several publications that discuss different issues associated with the time arrow. 

“Quantum Weak Invariants: Dynamical Evolution of Fluctuations and Correlations” by Shi and Abe [3] is dedicated to irreversible processes and their invariants in quantum mechanics. The microscopic processes are examined using the quantum mechanical interpretation of the physical reality and, at the same time, involves temporal asymmetry, which is conventionally linked to increasing entropy. 

“Some Aspects of Time-Reversal in Chemical Kinetics” by U. Maas [4] deals with time-directional relaxation of chemical kinetics into low-dimensional manifolds. This is determined by microscopic properties of chemical kinetics resulting in macroscopic irreversibility of reacting systems and forward-time convergence to states of partial or full equilibrium. 

Coretti, Rondoni and Bonetta [5] derive the fluctuation relation for a dissipative systems subject to parallel electric and magnetic fields for the first time. Unlike earlier work, the authors used a generalized time-reversal mapping so that it was not necessary to reverse the sign of the field to generated trajectory pairs. The results are tested using careful nonequilibrium molecular dynamics simulations. 

The article “Supernova and the Arrow of Time” by Abarzhi et al. [6] examines the Rayleigh–Taylor (RT) and Richtmyer–Meshkov (RM) instabilities developing on very large scales during explosions of stars. This phenomenon involves mixing connecting astrophysical and atomic scales and is strongly time-directional. 
